# Burden of traumatic spine fractures in Tehran, Iran

**DOI:** 10.1186/1471-2458-11-789

**Published:** 2011-10-11

**Authors:** Maziar Moradi-Lakeh, Mohammad R Rasouli, Alexander R Vaccaro, Soheil Saadat, Mohammad R Zarei, Vafa Rahimi-Movaghar

**Affiliations:** 1 Dept. of Community Medicine, School of Medicine, Tehran University of Medical Sciences, Tehran, Iran; 2 Sina Trauma and Surgery Research Center, Tehran University of Medical Sciences, Tehran, Iran; 3 Thomas Jefferson University and the Rothman Institute, Philadelphia, PA 19107, USA; 4 Research Centre for Neural Repair, University of Tehran, Tehran, Iran

## Abstract

**Background:**

The Disability-Adjusted Life Year (DALY) was designed by the World Health Organization (WHO) to measure, compare, and analyze the burden of various diseases. To the best of our knowledge, this is the first study on the assessment of burden of traumatic spinal fracture (TSF) in an Iranian community. We estimated burden of TSF includes both isolated (iTSF) and associated injuries related to traumatic spinal fractures (aTSF) in Tehran, the capital of Iran, for the year 2006-2007 using DALYs.

**Methods:**

Burden of TSF was estimated based on information provided by the national data on Iranian trauma, data from the WHO, and literature data using disease modeling (DISMOD). Incidence of TSF and associated injuries were obtained from two population based studies and National Trauma Data Bank in Iran, while duration, and relative risk of mortality (RRM) were obtained from WHO data and the literature. The incidence, duration, and relative risk of mortality (RRM) were used to calculate DALY for TSF. To calculate DALY, the years of life lost because of premature mortality (YLL) were added to the number of years lost because of disability (YLD). DALYs were calculated separately for both iTSF and aTSF. In-hospital YLD and post-hospital YLL for iTSF and in-hospital YLL and YLD were calculated for aTSFs.

**Results:**

TSF incidence was 16.35 (95%CI: 3.4-48.0) per 100,000. The incidence of TSF in males was more than twice that of females. The largest DALYs were seen in 15-29 years. The highest burden of associated injuries of TSF was related to spinal cord and head injury. DALYs for aTSF were estimated to be 2496.9 years (32.0 DALY/100,000 population). The YLD and YLL were almost similar. Total DALY for iTSF and aTSF was 2568.9 years (32.92 DALY/100,000 population). Based on the risk extracted from the literature, post-hospital increased risk of mortality was increased by 1318 DALY (16.89 DALY/100,000 population).

**Conclusion:**

This study showed a considerable burden for TSFs mainly due to associated injuries and increased lifelong RRM in patients with TSF.

## Background

Traumatic spine fracture (TSF) is commonly considered to cause short-term disability,[1] but may also result in long-term disability; its role as a potential cause of disability and/or death is usually neglected. TSF can occur either as an isolated lesion (iTSF) or associated with other injuries (aTSF) such as to the spinal cord, head, extremities, and other organs. Several studies have suggested individuals with iTSF over time may have a higher risk of mortality compared to the normal population [2,3]. In addition, aTSF may be associated with permanent disability [4]. Although the incidence of TSF increases in the elderly population,[5-8] TSF mainly involves young persons,[9] which may increase the overall burden of this disease.

The disability-adjusted life year (DALY) was designed by the World Health Organization (WHO) to measure, compare, and analyze the burden of various diseases [1]. A DALY is equal to the loss of one year of "healthy" life. To the best of our knowledge, there is no previous study on the measurement of TSF's affect on the Iranian community using the DALY. TSF does not have a considerable allocation of the total burden of injuries and diseases, but there are special groups of health care providers who care for TSF patients and their families who would have interest in TSF's burden on society. We searched PubMed and two Iranian databases, Iranmedex and Scientific Information Database (SID), with no similar studies found. This study aimed to estimate the magnitude and distribution of TSF including both iTSF and aTSF in Tehran from March 2006 to March 2007 using DALY. The results of this study could identify key opportunities for advancement in public health policies and research.

## Methods

The 2006 census was used to determine Tehran's urban population(~ 8 million), age, and sex distribution [10]. The International Classification of Diseases, 10th revision (ICD-10) codes of S12.0, S12.1, S12.2, S12.7, and S12.9 for cervical vertebral fractures, S22.0, and S22.1 for thoracic and S32.0 and S32.7, S32.8 for lumbar vertebral fractures were used to identify patients with TSF in the National Trauma Data Bank (NTDB) [11]. The following codes were totally or partially included but happened to have zero cases in our dataset: S18, S19, S22.9, S29.7, S29.9, S32.8 and S39.9. The details of the NTDB design and development have been previously described [11]. In brief, the NTDB is a database on more than 16,000 trauma patients that was collected from 1999 to 2004 in 8 major cities of Iran including six hospitals in Tehran. Average annual values for age and sex distribution (Table 1), in-hospital outcome (death vs. survival), and associated injuries of all TSF patients were also extracted from the databank.

**Table 1:** The distribution of total traumatic spine fracture in different ages and sexes in 619 patients from National Trauma Data Bank in Iran, 1999-2004

	< 1	1-4	5-14	15-24	25-34	35-44	45-54	55-64	65-74	75≤	Total
**Female** **Count (**%)	0 (0)	1 (0.16)	11 (1.78)	25 (4.04)	41 (6.62)	31 (5.01)	31 (5.01)	34 (5.49)	17 (2.75)	4 (0.65)	195 (31.5)

**Male** **Count (**%)	0 (0)	1 (0.16)	16 (2.58)	112 (18.09)	108 (17.45)	90 (14.54)	40 (6.46)	30 (4.85)	20 (3.23)	7 (1.13)	424 (68.5)

**Total** **Count (**%)	0 (0)	2 (0.32)	27 (4.36)	137 (22.13)	149 (24.07)	121 (19.55)	71 (11.47)	64 (10.34)	37 (5.98)	11 (1.78)	619 (100)

**Figure 1:**
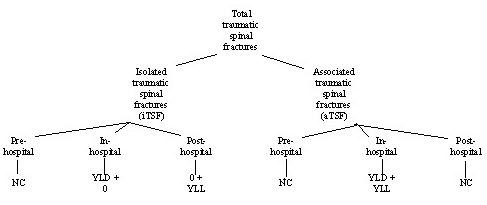
**Summary of calculation of disability-adjusted life year (DALY) for various types of traumatic spine fractures**. Footnote of the Figure 1: NC: Not calculated. YLL: Years of Life Lost because of premature mortality. YLD: Years Lost because of Disability.

In the present study, TSF cases were divided into two groups as follows; iTSF and aTSF (Figure 1); DALY was calculated for each group separately. Total DALY was achieved by summing the calculated DALYs for each group. There were a total of 619 TSF patients, 354 iTSF and 265 aTSF patients. Cervical, thoracic, and lumbar fractures were included in the study and sacrococcygeal and pelvic fractures were excluded.

The method of DALY calculation for TSF was similar to that which was used to determine burden of spinal cord injury (SCI) in Tehran [12]. In brief, DALY was calculated by summation of two components: years of life lost due to premature mortality (YLL) and number of years lost because of disability (YLD). We used DisMod (disease-modeling) software,[1] which provides an internally consistent set of epidemiological indices including incidence, prevalence, remission, duration, mortality, case fatality, and relative risk of mortality for diseases and injuries. For this purpose, the data on population structure, general mortality rates and at least three of the above mentioned epidemiological indices are necessary.

Disability weights (DWs) for TSF and other important coincident injuries were considered equal to the global burden of disease (GBD) for short and long-term disabilities; which was 0.266 for iTSF [13,14]. In the present study, we included only SCI, head, and extremity injuries in the calculation. Age-specific incidence rates were used to calculate YLD for iTSF. Short-term mortality of iTSF was considered as zero. To calculate YLD for aTSF, we estimated common DWs and duration of each coincident injury by multiplying average duration of each disability by common DW. Co-morbid DWs were calculated as previously described [15].

### Discount rate and age-weighting

Discount rate means that future life years are assigned less value than those lived today, typically assigned as 3%. We used standard age-weights for estimating DALYs[16]

### Parameters were used for calculation of DALY

#### Incidence

To estimate the incidence of TSF for two consecutive Iranian calendar years (21.3.2005-20.3.2006 and 21.3.2006 to 20.3.2007), two population based studies were performed in September 2007 and 2008 in Tehran [17]. In brief, cluster random sampling was used and structured interviews were performed with participants to detect history of spine fracture. All cases with positive history were evaluated by a specialist to document the spine fracture. The annual incidence was estimated by dividing the number of TSF cases that occurred during these two years by the total participants in two years. Totally, 3 new cases of TSF (C1- L5) were found in 18,346 person-years that provided an incidence of 16.35 (95%CI: 3.4-48.0) per 100,000. TSF incidence was 21.29 and 11.17 per 100,000 for males and females, respectively. All suspected cases of spinal fractures had been evaluated by their medical records and documents. There was a list of deceased among the household survey, but none of them were suspected to have a TSF. The other concerns are common shortages of estimating incidence based on cross-sectional surveys and interviews with current residents. Historically there is underestimation of incident cases in a population-based survey versus hospital-based data. Although there was a low number of cases, shown by wide 95% confidence interval the incidence of spinal cord injury (SCI) found in almost half of these TSFs was compatible with our previous publication on burden of SCI [17].

### Duration of disease

Global Burden of Disease (GBD) and studies supported by the WHO, suggested 0.14 year or 51 days as the duration of disease for TSF [1,14]. Therefore, 51 days was considered as the duration of disease for TSFs; in cases of aTSF, duration of the other associated injuries such as injury to the brain, spinal cord or femur fracture were considered as well. There are some evidences for lifelong additional risk of mortality after TSF.

### Relative risk of mortality (RRM)

According to an unpublished report of death registry by the Iranian ministry of health, the crude death rate of the country is estimated as 3.6 per 1000 persons. RRM was estimated by summation of pre-hospital, in-hospital, and post-hospital mortalities (Figure 1). Among 25 cases who expired in the hospital, 2 cases had been categorized as iTSF. It was assumed that these 2 patients had associated injuries that had been missed or not been recorded as it is highly unlikely that isolated TSFs would result in death. Thus, in the iTSF group, pre-hospital and intrahospital mortality were not included in the calculation resulting in a YLL of zero. There are several papers that showed some additional risk for delayed mortality following TSF [3,4,18]. In the study of Puisto and colleagues, a lifelong RRM of 1.33 for females and 1.43 for males following TSF was reported. This RRM was mainly due to cancers or respiratory diseases, even after exclusion of metastatic fractures at the time of TSF occurrence [19]. We considered post-hospital mortality based on these studies. However, Victorian and other studies supported by WHO did not calculate any burden beyond 51 days (0.14 year). Post-hospital YLD was estimated to be zero, because there was no definite evidence for continuous pain or disability after 51 days.

An additional consideration was calculation of DALY for associated injuries in TSF. We did not consider the mortalities of associated injuries during pre- or post-hospital intervals. We assumed that all pre and post-hospital mortalities were definitely attributable to associated injuries not TSF and should be considered within the burden of their own injuries.

The ethical committee of "Sina Trauma and Surgery Research Center of Tehran University Medical Sciences" approved our study. Patients did not take Part in the study and. Thus, they did not give informed consent.

## Results

### Isolated traumatic spine fractures (iTSF)

The estimated sex-age specific incidence of 354 cases of iTSF is demonstrated based on disease modeling in Table 2. Burden of iTSF consisted of two components, in-hospital YLD and post-hospital YLL. Considering no weight for residual disability after the first 51 days, the burden of iTSF in post-hospital phase is due to additional mortalities as described above (Table 3). Post-hospital YLL was calculated by considering the RRM of 1.33 and 1.43 for females and males respectively. Total post-hospital DALY for iTSF in this phase was 1318 years, 427 years for females and 891 years for males, with a female to male ratio of 47.92%. In-hospital DALY for iTSF was 72 years, consisting of 24 years for females and 48 years for males.

**Table 2:** The estimated sex-age specific incidence of isolated traumatic spine fracture per 100,000 of the general population based on disease modeling based on two population studies in two consecutive years (21.3.2005-20.3.2006 and 21.3.2006 to 20.3.2007)

Age	Males	Females
**0**	0.62	0.71

**1-4**	0.92	0.92

**5-9**	2.41	1.97

**10-14**	3.75	2.78

**15-19**	7.52	3.13

**20-24**	13.05	3.76

**25-29**	14.66	4.78

**30-34**	15.27	6.05

**35-39**	15.85	6.48

**40-44**	14.53	6.68

**45-49**	11.91	7.53

**50-54**	10.7	9.2

**55-59**	11.57	11.93

**60-64**	12.7	15.36

**65-69**	13.48	15.11

**70-74**	12.81	11.25

**75+**	9.95	6.24

**Total**	10.89	5.61

**Table 3:** Post-hospital disability-adjusted life year (DALY) for isolated traumatic spine fractures (iTSF) by considering the relative risk of mortality of 1

	Males			Females			Both male and female patients		
***Age***	**Population**	**DALY**	**DALY/10^5^**	**Population**	**DALY**	**DALY/10^5^**	**Population**	**DALY**	**DALY/10^5^**

**0-4**	234,131	-	-	222,800	-	-	456,931	-	-

**5-14**	514,665	2	0.39	490,990	-	-	1,005,655	2	0.2

**15-29**	1,348,606	48	3.56	1,291,414	5	0.39	2,640,020	53	2.01

**30-44**	948,147	119	12.55	910,440	15	1.65	1,858,587	134	7.21

**45-59**	589,581	223	37.82	573,833	46	8.02	1,163,414	270	23.21

**60-69**	198,380	180	90.73	181,438	67	36.93	379,818	247	65.03

**70+**	152,909	318	207.97	146,549	295	201.3	299,458	612	204.37

**Total**	3,986,419	891	22.35	3,817,464	427	11.19	7,803,883	1,318	16.89

### Associated traumatic spine fractures (aTSF)

The frequency and percentage of the main associated injuries (aTSF) with TSF is shown in Table 4. Although superficial injuries were the most common associated injuries, there was no affect on disability. Thus, the YLD became zero and superficial injuries were subsequently deleted. As Table 4 shows, open wounds are one of the most common associated injuries, but have one of the lowest YLDs compared with other associated injuries such as head traumas, skull fractures, SCIs, and femur fractures (Table 5). In the 25 deceased patients with aTSF there were 9 patients with intracranial hemorrhage, 2 skull fractures, 6 spinal cord injuries (SCI), 4 TSFs with associated dislocations, 1 tracheolaryngeal crush, 1 open wound, and 2 unknown injuries. The highest burden of associated injuries of TSF was related to SCI and head injury including skull fracture and brain injury with or without hemorrhage. Mean YLD per case for each associated injury was demonstrated in Table 5. The highest DALY was seen in 15-29 years followed by 30-44 years. DALY for aTSF was estimated to be 2496.9 years (32.0 DALY/100,000 population) (Table 6). The burden of aTSF was 843.69 years for females and 1725.1 years for males; female: male ratio was 48.91%.

**Table 4:** Frequency and percent of associated injuries to total traumatic spine fracture and in associated traumatic spine fractures (aTSF)

	Frequency	Percent in total TSF	Percent in aTSF
SCI	33	5.33	12.45

Open wound	40	6.46	15.09

Open wound plus skull Fx	20	3.23	7.55

Skull Fx	6	0.97	2.26

Brain injury	20	3.23	7.55

ICH + Skull Fx	5	0.81	1.89

Femur Fx	7	1.13	2.64

Tibia, Fibula or Ankle Fx	28	4.52	10.57

Foot Fx	13	2.1	4.91

Femur and Upper limb Fx	3	0.48	1.13

Forearm + Leg Fx	4	0.65	1.51

Femur + Leg Fx	1	0.16	0.38

Humerus Fx	9	1.45	3.4

Forearm Fx	20	3.23	7.55

Hand Fx	6	0.97	2.26

Brain injury + Limb Fx	31	5.01	11.7

Fx + Dislocation	19	3.07	7.17

Total aTSF	265	42.81	100

Fx: Fracture

ICH: Intracranial hemorrhage

**Table 5:** Mean Years Lost because of Disability (YLD) per case for each associated injury and mean age for associated Traumatic Spine Fracture (aTSF)

	Mean age (yr)	YLD according to the type of disability	
		
		Long-term	Short-term
SCI	37	17.332	-

Open wound	37	-	0.039

Open wound plus skull Fx	35	8.409	0.072

Skull Fx	28	8.408	0.055

Brain injury	41	10.362	0.055

ICH + Skull Fx	26	15.232	0.081

Femur Fx	43	6.554	0.075

Tibia, Fibula or Ankle Fx	36	-	0.055

Foot Fx	44	-	0.041

Femur and Upper limb Fx	33	7.221	0.087

Forearm + Leg Fx	39	-	0.067

Femur + Leg Fx	59	4.623	0.087

Humerus Fx	46	-	0.052

Forearm Fx	44	-	0.052

Hand Fx	43	-	0.042

Brain + Limb Fx	33	-	0.068

Dislocation	36	-	0.038

Fx: Fracture

ICH: Intracranial hemorrhage

The Years Lost because of Disability (YLD) attributed to the associated injuries were calculated by multiplying total number and YLD per case (considering the weights of Short-term and Long-term disabilities)

**Table 6:** In-hospital DALY for associated Traumatic Spine Fracture (aTSF)

	Males			Females			Both male and female patients		
***Age***	**Population**	**DALY**	**DALY/10^5^**	**Population**	**DALY**	**DALY/10^5^**	**Population**	**DALY**	**DALY/10^5^**

**0-4**	234,131	2.5	1.07	222,800	2.7	1.21	456,931	5.2	1.14

**5-14**	514,665	38.8	7.54	490,990	29.4	5.99	1,005,655	68.1	6.77

**15-29**	1,348,606	389.5	28.88	1,291,414	120.0	9.29	2,640,020	509.4	19.3

**30-44**	948,147	275.5	29.06	910,440	109.9	12.07	1,858,587	385.4	20.74

**45-59**	589,581	89.1	15.11	573,833	90.1	15.7	1,163,414	179.2	15.4

**60-69**	198,380	27.2	13.71	181,438	33.7	18.57	379,818	60.9	16.03

**70+**	152,909	12.9	8.44	146,549	12.1	8.26	299,458	25.0	8.35

**Total**	3,986,419	835.4	20.96	3,817,464	397.8	10.42	7,803,883	1233.3	15.8

### Total burden of TSF

To calculate the total burden of TSF, DALY for iTSF was added to the DALY for aTSF, which provided a total DALY of 2568.9 years (32.92 DALY/100,000 population). There are an additional 1318 years DALY (16.89 DALY/100,000 population) for post-hospital increased risk of mortality based on literature papers (Table 7).

**Table 7:** Components of burden of traumatic spine fracture and estimated value

		Males		Females		Total	
		
		YLD	YLL	YLD	YLL	YLD	YLL
Pre-hospital	Isolated	0	0	0	0	0	0

Pre-hospital	Associated	NR	NR	NR	NR	NR	NR

In-hospital	Isolated	48	0	24	0	72	0

In-hospital	Associated	841.7	835.4	421.9	397.8	1263.6	1233.3

Post-hospital	Isolated	0	891	0	427	0	1318

Post-hospital	Associated	NR	NR	NR	NR	NR	NR

Total	Isolated	48	891	24	427	72	1318

Total	Associated	841.7	835.4	421.9	397.8	1263.6	1233.3

NR = Not related and have to be discuss in the related subject of brain, spinal cord, femur and other injuries

## Discussion

To the best of our knowledge, this study is the first to separately calculate DALY for TSF. There are two points that should be noted in the interpretation of these results. The first point is related to the considerable calculated burden for aTSFs compared to the estimated DALY for iTSFs. The associated injuries accounted for the additional burden of aTSF compared to iTSF.

The second and more important point is related to the discrepancy between our calculated DW for iTSF by GBD, versus other investigations [3,4,13,14,19] In Victorian and GBD studies, post-hospital DALY for TSF probably included the burden of chronic disease such as cancer and respiratory diseases. In the present study, we did not add the total post-hospital DALY for TSF, 1318, with burden of TSF, 2496.9.

This study differs from previous studies in the estimation of the endpoint of the sequelae of a disease. GBD evaluated a window of 0.14 year (51 days) disability following TSF [2]. If DALY were calculated based on GBD data, the DALY for iTSF would be 72 years. However, there are several studies that demonstrate that patients with TSF have higher mortality rate in long-term compared to general population [3,4,19]. In a 5-year observational cohort study, a total of 7753 randomly-selected individuals (2187 men and 5566 women) aged ≥ 50 years from across Canada were evaluated [3]. In this large sample, risk of mortality in participants with incidental TSF was compared with participants without TSF. The study revealed that the risk of mortality increased in those patients who had a TSF in the second year (adjusted hazard ratio [HR]: 2.7, 95% confidence interval [CI] 1.1-6.6). Among women, the risk of death was increased for those with a vertebral fracture during the first (adjusted HR: 3.7, 95% CI: 1.1-12.8) or the second year of follow-up (adjusted HR: 3.2, 95% CI: 1.2-8.1) [3]. Center et al. showed a higher mortality compared to general population in the first year after all major fractures in women and men older than 60 years. Following spine fracture, age standardized mortality ratios were 1.66 (95%CI: 1.51-1.80) 2.38 (95%CI: 2.17-2.59) in males and females [4]. Puisto and colleagues demonstrated that spine fracture significantly predicted total mortality due to increasing cancer and respiratory deaths. The increased risk of cancer death persisted even when those with a history of cancer were excluded to eliminate the effect of metastatic fractures. During all life, RRM increased for both sexes as it was 1.33 for females and 1.43 for males [19]. According to these facts, the aforementioned RRM in the later study was considered in calculation of burden of TSF which added 1318 additional years to the previously calculated 72 years.

The importance of associated injuries in the mortality of TSF has been shown in several studies. The uncommon traumatic cervical spine fractures and dislocations were studied in 227 consecutively treated children and juveniles (1 to 17 years of age). There were 19 deaths (8.4%), all of which were associated with injuries at the C4 level or higher. Of the 11 patients with atlanto-axial fracture or dislocation, all died soon after the injury. All had an unstable fracture and cord transection that resulted in cardiorespiratory collapse. Injuries at the vertebral levels C1, C2, C3, and C4 were associated with fatality rates of 17%, 9%, 4.3%, and 3.7%, respectively [20]. In our study 6 of the 25 deceased adult patients with aTSF had SCI.

Regarding other associated injuries, we did not find a correlation between thoracoabdominal injuries and increased rate of mortality. Although the patients were severely injured, it was mainly due to increased incidence of associated thoracic injuries. No significant difference in mortality was observed [21].

This study had several limitations. Data for the population of Tehran was available at 2006, but the data for incidence was obtained from a wider period of time and the data for sex, age, and associated injuries was related to 1999-2004. Different types of vertebral fractures have the same DW; therefore these were not separated. Major associated injuries were used and some minor associated injuries were deleted. We believe that the annual incidence has not changed significantly in two consecutive years. The age-sex structure of people with spinal fracture is influenced by different risk factors among sex and age subgroups. During the recent decade, there was no recognized change in the pattern of risk factors in sex-age subgroups. Therefore, the authors believe that these assumptions have no significant impact on the estimation of TSF burden. We do not expect to have different age-sex pattern of spinal fractures among large Iranian cities.

Patient inflow of third-level hospitals in Tehran include many patients from other cities., so the patient population that is represented by the six hospitals in Tehran does not exactly represent the population of Tehran. However, we only used the age-sex pattern from that datum, not the incidence of fracture.

Osteoporosis is a known risk factor for fractures including TSF. Burden of osteoporosis in Iran has been estimated elsewhere [22].

In the cases of aTSF, some part of the estimated burden is attributable to associated injuries not to TSF. Also underlying conditions can increase burden of fracture both in iTSF and aTSF. This study showed a higher proportion of TSF burden was related to the associated injuries than iTSF.

## Conclusions

Our study was the first investigation that attempted to calculate the burden of TSF. In a localized Tehran environment, the present study showed a considerable burden for TSF which is mainly due to simultaneous associated injuries and lifelong higher RRM in subjects with TSF. The results of TSF burden will be used to advise policy makers, prioritization of preventive measures, support the evaluation of interventions, and provide guidance on the degree of impairment and disability following specific types of TSF. Considering the high proportion of associated injuries in TSF, it is recommended that policy makers prepare preventive strategies to reduce the number and severity of aTSF.

## Competing interests

The authors declare that they have no competing interests.

## Authors' contributions

MML carried out the analysis, participated in design and drafting the manuscript. MRR carried out the systematic review and provided input data for analysis. SS provided data on incidence of TSF in Tehran. MRZ provided data on NTDB. ARV edited the paper and technical consults for TSF modeling. VRM carried out the design, participated in coordination, analysis and drafting the paper. All authors read and approved the final manuscript.

## Pre-publication history

The pre-publication history for this paper can be accessed here:

http://www.biomedcentral.com/1471-2458/11/789/prepub
